# Time to hypertension development among people living with HIV in South Africa: A longitudinal analysis of the National Income Dynamics Survey (NIDS)

**DOI:** 10.1016/j.heliyon.2021.e07750

**Published:** 2021-08-09

**Authors:** Motlatso Godongwana, Nicole De Wet-Billings

**Affiliations:** a Demography and Population Studies, Schools of Social Sciences and Public Health, Faculties of Humanities and Public Health, University of the Witwatersrand, Johannesburg, South Africa; b Perinatal and HIV Research Unit, Chris Hani Baragwaneth Hospital, Johannesburg, South Africa

**Keywords:** Hypertension, HIV, Chronic conditions, Comorbidities, Morbidity

## Abstract

**Introduction:**

There is an increased risk of hypertension among people living with HIV (PLWH). Older age has been associated with a higher risk of chronic conditions. In this study, we assess the time taken before adults living with HIV develop hypertension and explore the factors associated with hypertension diagnosis among PLWH.

**Methods:**

Retrospective analysis on (n = 95 701) HIV positive adults from the longitudinal survey data from the National Income Dynamics Survey (NIDS) in South Africa was performed. The adults (18–75 years) were followed in order to determine the age of hypertension risk. Kaplan Meier survival estimates were used to show time to diagnosis. Multivariate cox regression model was used to determine the factors associated with hypertension diagnosis.

**Results:**

10.5% had HIV and hypertension at the start of the NID survey (wave 1:2008). Of the remaining (n = 85 569), over 75% aged 30–46 were at risk of developing hypertension. Thereafter the risk of hypertension comorbidity begins to decrease after the age of 45. In other words, the risk of hypertension began to reduce once the adults living with HIV turned 45 years old. There was no significant association between the development of hypertension comorbidity and the other demographic, socio-economic and health characteristics assessed.

**Conclusion:**

Young adults living with HIV are also at risk of hypertension. HIV infected persons need to routinely screen for chronic diseases and started on treatment timeously.

## 1 Introduction

People living with HIV (PLHIV) are at an increased risk of developing hypertension [1, 2, 3]. The global prevalence of hypertension in HIV infected adults is about 35% [4]. Similarly, several studies in the sub-Saharan region have found levels of HIV infected persons living with hypertension to range between 17% - 30% [5, 6, 7]. However, some studies have reported a prevalence of over 40% [8]. In South Africa, about 22% of HIV positive adults have been found to be living with hypertension [9]. The high levels of adults living with HIV and hypertension is concerning because it is associated with increased morbidity and mortality [10]. The comorbidity caused by HIV and hypertension further leads to overburdened health systems, increased medical costs for patients and a decrease in overall quality of life [11].

Among HIV uninfected persons, hypertension commonly occurs among females [12]. However, factors associated with the risk of hypertension among HIV positive persons include male, older age, prolonged duration of HIV infection, on Antiretroviral Treatment (ART); family history of hypertension, high viral load and body mass index [6, 9, 13]. About 20% of the adult population (15–49 years) in South Africa is HIV positive [14]. Several studies suggest that HIV positive adults older than 40 years old are at most risk of developing hypertension [7, 9, 13, 15, 16]. In countries most affected by HIV such as South Africa, the highest rate of HIV is among adolescent girls and young women (AGYW) aged 15–24 years old [14]. With an increased risk of hypertension as PLHIV grow older and in the context of existing literature, some of these AGYW and adults living with HIV will be at risk of hypertension only after the age of 40. To our knowledge, there is no study that has investigated the time taken before adults living with HIV develop hypertension.

The objective of this study is to [1] assess time (age in years) to the development of hypertension among adults already living with HIV and [2] understand the factors associated with this comorbidity.

## 2 Methods

### 2.1 Study design and setting

This study is a retrospective analysis of longitudinal survey data from the National Income Dynamics Survey (NIDS) in South Africa. Permission to use the NIDS data was obtained from Data First. South Africa is selected as a country of analysis because of the high burden of HIV (20%) and adults living with both HIV and hypertension (22%) [14]. The NIDS is conducted in all nine provinces in South Africa, every two years. The first wave of the study began in 2008. To date, four additional waves have occurred between 2010 and 2017 [17]. The time points of interest in this study are waves 3 (2012) and waves 5 (2017). The NID surveys are administered using three main types of questionnaires namely: The Household, Adult, Children and the Proxy Questionnaire [17]. To obtain all of the variables of interest including HIV and hypertension status, the adult data file was merged with the child and household data files.

### 2.2 Study population and sample size

We restricted our analysis to adults 18–75 years diagnosed with HIV at wave 1. The total weighted adult sample was 5,961,149. After restricting the sample to HIV positive adults only, a sample of 95,701 HIV positive adults remained at wave 1. Of these, about 10.5% were diagnosed with hypertension. This study assessed 85,569 HIV positive adults that remained undiagnosed with hypertension at wave 1. We retrospectively followed these adults to waves 3 and 5 in order to determine the time (age in years) taken before they developed hypertension.

### 2.3 Variable description

Only HIV positive adults were included in the study and followed through to waves 3 and 5. The outcome variable in this study is hypertension diagnosis. The outcome is dichotomous and has two categories [1] Yes and [2] No. The “Yes” represents HIV positive adults who would have developed the outcome and “No” represents those that did not develop hypertension”. The demographic, socio-economic and health behaviour predictor variables that are used in this study include *gender, race, marital status, religion, place of residence, employment status, level of education, household income, tobacco and alcohol use, frequency of exercise, perceived health and last doctor's consultation.*

### 2.4 Analysis

We conducted all analysis using STATA version 14.0. Cross tabulations were used to describe the characteristics of the HIV positive adults living with hypertension at wave 1 of the NIDS survey. The data was reformatted in a person-period data file in order to allow for longitudinal data analysis and address the issue of right censoring. Right censoring refers to a case whereby an HIV positive adult would not have developed hypertension by the end of the study period or drops out of the study before the end of the study. A major strength of the Cox proportional hazard model of analysis is that it addresses the issues of right censoring by ensuring that persons who dropped out or did not develop the outcome of interest are kept in the study but excluded from the analysis in the regression. To do this, the longitudinal data is set in person-period data mode in STATA. By applying Cox proportional procedures, both censored and uncensored observations were accounted for and the results reported in this study represents the findings after censoring has been accounted for.

After applying the Cox model, Kaplan Meier survival estimates were used to assess the time (age in years) in which the adult sample developed hypertension. The survival estimates were created using the model equation incorporated in STATA:

$$S\left(t_{i}\right)=\underset{t_{i}\leq t}{∐}\left(1-\frac{d_{i}}{n_{i}}\right)$$

Where:

*S(ti)* = estimated survival probability at time *t*
*ni* = number of people at risk of the event of interest at the beginning of time period *t*_*i*_
*di* = number of diseases that occurred at time *t*_*i*_

The following STATA commands were then executed to run the graphs.

*stset age, failure(Diagnosed3)*
*sts graph if age ≤ 75, xlabel (18(7)75)*
*sts list*

In this study, the time period is measured from wave 1, which represents the time in which the participants entered the study with a positive HIV result and waves 3 and 5 which represent the time in which these adults would/would not have developed hypertension. The Kaplan Meier provide a graphical depiction of the time to the occurrence of hypertension among persons with HIV at waves 3 and 5. We then used the cox hazard regression model to assess the effect of each of the predictor variable on the development of hypertension at wave 3. The level of significance was set at p < 0.05. The adjusted and unadjusted results are presented and interpreted as hazard ratios.

## 3 Results

The results in Table 1 show the percentage distributions of hypertension comorbidity at wave 1 as well as the hazard of hypertension among PLWH by the different demographic, socio-economic and health characteristics at waves 3. At wave 1 of the NID survey, 10 131 (10.5%) adults living with HIV were diagnosed with hypertension comorbidity. Of these adults, the majority (58.1.8%) were above the age of 35 years. At wave 1, almost all of the HIV positive adults that were additionally diagnosed with hypertension in this study were female (97.1%), the majority never married (67.1%), all were Christian and largely African (88.2%). A high percentage (76.6%) resided in urban areas of South Africa, were unemployed (88.2%) and over half (64.8%) came from households with a below average household income. Interestingly, over 70% had received some education and a large number reported to neither smoke nor drink alcohol. In addition, most of these adults struggled with performing regular exercise (63.7%) and almost 70% reported to have poor health. A large proportion (97.1%) of these adults had consulted with a doctor in the last 30 days.

**Table 1:** Percentage distribution and adjusted hazard ratios of hypertension comorbidity among PLWH by individual characteristics, NIDS waves 1 & 3 South Africa.

Demographic variables	Adjusted Hazard ratios [95% Confidence Intervals]	P – values (p= <0.05)	Diagnosed N = 10 131	Undiagnosed N = 85 569	Total N = 95 701
**Age**					
≤35 years (%)			4 244.5 (41.89)	28 053.1 (32.78)	32 269.3 (33.7)
>35 years (%)	-		5 887.2 (58.11)	57 516.5 (67.22)	63 403.8 (66.2)
**Gender**					
Female (%)	RC		9 843.4 (97.15)	61 064.9 (71.36)	70 908.5 (74.0)
Male (%)	0.22 (0.00–5.60)	0.36	288.3 (2.85)	24 504.7 (28.64)	24 793.1 (25.9)
**Marital status**					
Never married (%)	RC		6 807.2 (67.19)	52 543.5 (61.40)	59 350.7 (62.0)
Married (%)	1.85 (0.07–47.64)	0.71	3 324.4/(32.81)	33 026.2 (38.60)	36 350.7 (37.9)
**Religion**					
Other religion (%)	RC		0 (0)	5 610.4 (6.56)	5 610.4 (5.8)
Christian (%)	0.03 (0.00–2.46)	0.12	10 131.7 (100)	79 959.2 (93.44)	90 091.0 (94.1)
**Race**					
Other (%)	RC		1 186.4 (11.71)	423.4 (0.49)	1 609.9/95 701 (1.6)
African (%)	2.31 (0)	1.00	8 945.3 (88.29)	85 146.2 (99.51)	94 063.2/95 701 (98.3)
**Place of residence**					
Rural (%)	RC		2 364.0 (23.33)	54 119.6 (63.34)	56 563.6 (59.0)
Urban (%)	0.04 (0.00–2.64)	0.13	7 767.7 (76.67)	31 370.1 (36.66)	39 137.9 (40.9)
***Socio-economic status***					
**Employment status**					
Unemployed (%)	RC		8 945.3 (88.29)	78 488.3 (91.72)	87 433.7 (91.3)
Employed (%)	0.124 (0.00–4.74)	0.26	1 186.4 (11.71)	7 081.4 (8.28)	8 267.8 (8.6)
**Household income**					
B/low average (%)	RC		6 573.2 (64.88)	66 968.4 (83.08)	73 541.7 (81.0)
Average (%)	194.39 (0.52–71797.24)	0.08	3 558.5 (35.12)	13 637.8 (16.92)	17 196.3 (18.9)
**Education level**					
No education (%)	RC		2 805.3 (27.69)	18 562.6 (21.69)	21 368.0 (22.3)
Some education (%)	10.62 (0.07–1514.991)	0.35	7 326.4 (72.31)	67 007.0 (78.31)	74 333.5 (77.6)
***Health Behaviour***					
**Tobacco use**					
No (%)	RC		8 236.5 (81.29)	67 586.4 (78.98)	75 822.9 (79.2)
Yes (%)	1.25 (0.02–58.97)	0.90	1 895.2 (18.71)	17 983.3 (21.02)	19 878.5 (20.7)
**Alcohol use**					
Do not drink (%)	RC		7 090.5 (69.98)	66 119.2 (77.27)	73 209.8 (76.5)
At least 1nce a week (%)	3.89 (0.15–100.27)	0.41	3,041.2 (30.02)	19 450.5 (22.73)	22 491.7 (23.4)
**Exercise**					
Never (%)	RC		6 457.7 (63.7)	77 151.3 (90.16)	83 609.0 (87.3)
1nce a week (%)	5.14 (0)	1.00	3 674.0 (36.26)	8 418.4 (9.84)	12 092.4 (12.6)
**Perceived Health**					
Poor (%)	RC		7 019.6 (69.28)	69 085.4 (80.74)	76 105.0 (79.5)
Good (%)	0.002 (5.80–1.18)	0.05	3 112.1 (30.72)	16 484.3 (19.26)	19 596.4 (20.4)
**Last Dr consultation**					
Last 30 days (%)	RC		9 843.4 (97.15)	55 625.6 (65.01)	65 469.1 (68.4)
1month + ago (%)	10.28 (0.07–1361.40)	0.35	288.3 (2.85)	29 944.1 (34.99)	30 232.4 (31.5)

RC = reference category.

∗Significant (p= <0.05).

∗∗∗Some variables yielded no (-) or (0) results because they were not fit for the Cox Model.

**Figure 1:**
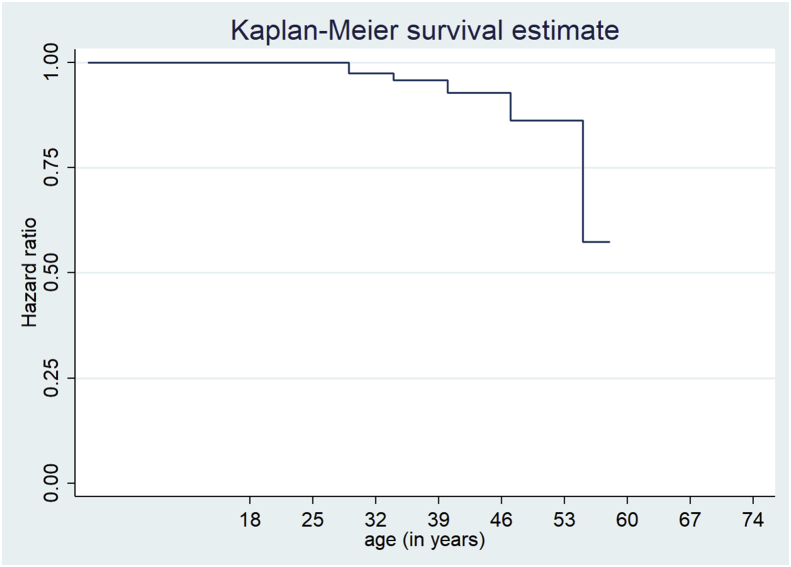
Hazard of hypertension comorbidity at wave 3, NIDS South Africa, 2012.

Figure 1 shows that at wave 3 of the NID survey, the risk of developing hypertension was highest among adults aged over 30 years old. The vertical x-axis represents the hazard ratio or risk and the horizontal x-axis indicate the age in which the HIV positive adults in this study became at risk of hypertension. As shown in Figure 1, before the age of 30, the risk of developing hypertension is low, as represented by the horizontal hazard line showing that the event of interest (hypertension) did not occur. From the age of 30, the hazard line begins to change, showing that the event of interest has occurred. Over 80% of HIV positive adults aged 30–46 years old were at risk of developing hypertension comorbidity. Thereafter the risk of hypertension comorbidity began to decrease with an increase in age after age 46. In other words, the risk of hypertension began to reduce once the adults living with HIV turned 46 years old. The hazard of developing hypertension comorbidity decreases significantly to about 55% from the age of 53.

**Figure 2:**
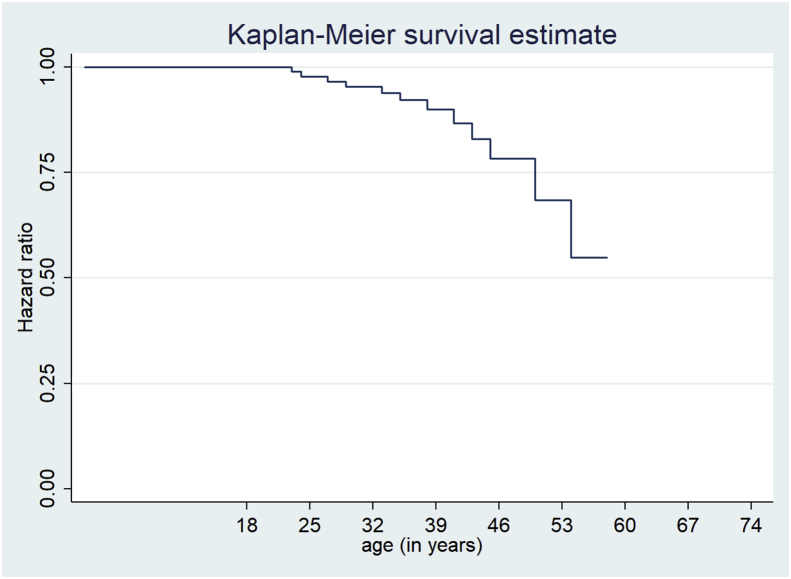
Hazard of hypertension comorbidity at wave 5, NIDS South Africa, 2017

At wave 5 of the NID survey (as depicted in Figure 2), the hazard of HIV positive adults developing hypertension comorbidity began from the age of about 23 years. Adults diagnosed with HIV were not at risk of hypertension before the age of 23, as represented by the horizontal hazard line. After the age 23 the hazard line begins to change, showing that the event of interest (hypertension) has occurred. The highest risk of hypertension comorbidity was between the ages 23–45. Over 75% of HIV positive adults within this age group were at risk of developing hypertension. Thereafter the risk of hypertension comorbidity begins to decrease with an increase in age after age 45. In other words, the risk of hypertension began to reduce once the adults living with HIV turned 45 years old. The hazard drops to about 70% at the age of about 48 and further down to approximately 52% by the age of 53. The hazard of developing hypertension comorbidity remained low from the age of 53.

A total of 85 569 adults living with HIV who were not diagnosed with hypertension at the end of wave 1 were then followed to waves 3 and 5 to assess whether they developed hypertension comorbidity over time. The results from the Cox proportional hazard model showing the risk (hazard ratios) of hypertension comorbidity by the varying characteristics at waves 3 are presented in Table 1. As depicted in the table, the hazard for hypertension comorbidity by age was not assessed in the Cox regression model because age is used as the risk time in the model and Kaplan survival estimates. In other words, the time to hypertension comorbidity is represented by the age at which the adults (aged 18–75 years old) living with HIV developed hypertension.

The results from the Cox model show that compared to the reference groups the risk of developing hypertension comorbidity was reduced by 78% among HIV positive adults that were male (HR:0.22). There was an increased risk of hypertension among adults that never married (HR:1.85). Compared to adults from other religious denominations, the hazard of hypertension comorbidity was reduced by 97% for Christian adults living with HIV (HR:0.03). The risk of hypertension was increased among African adults living with HIV (HR:2.31). Regarding place of residence, the risk of hypertension comorbidity was reduced by 96% for adults residing in an urban areas (HR:0.04). The hazard of hypertension comorbidity was also reduced by 88% for employed adults living with HIV compared to unemployed adults (HR:0.12). The study further found that adults living with HIV and earning an average household income had an increased risk to develop hypertension comorbidity compared to adults that had a below average household income (HR: 194.39). The hazard of hypertension and HIV comorbidity was increased among adults who smoked tobacco (HR:1.25), drank alcohol (HR:3.89), exercised at least once a week (HR:5.14) and had seen a doctor more than a month ago (HR: 10.28). Compared to adults with a perceived poor health, the hazard of hypertension comorbidity was decreased by almost 99% among adults with a perceived good health (HR:0.002). However, none of these demographic, socio-economic or health characteristics were significantly associated with the development of hypertension among adults living with HIV at the third wave in the model.

In the adjusted model, a borderline significant association was found between religion and hypertension development among PLWH. Results from the Cox model (Table 1) show that the hazard of developing hypertension was 1.12 higher for HIV positive Christians compared to other religions [OR:1.12; CI: 0.01376–1.04819]. Given the p-value set at p= <0.05, this result is insignificant but however may suggest that there is a trend towards the relationship between religion and the development of hypertension among adults living with HIV.

## 4 Discussion

This study found that younger PLWH are also at an increased risk to develop hypertension. The age of risk begins from 30 years. This is of notable importance given that young people, and particularly adolescent girls and young women (15–24 years) are the most affected by HIV/AIDS in the sub-Saharan region [18]. In other words, an adolescent diagnosed with HIV at the age of 15 will become at risk of hypertension at least 15 years after HIV diagnosis and will remain at risk for over 16 proceeding years.

An important contribution of this study is the result stating an increased risk of HIV and hypertension comorbidity among young adults. This finding shows the inverse of what has been found in previous studies. Most studies have found that the risk of hypertension for HIV positive adults is highest from the age of 40. In Zimbabwe, about 30% of HIV positive adults aged over 40 had hypertension [7]. A study in Ethiopia reported a high prevalence (54%) of hypertension in HIV positive adults older than 45 years [13]. Among adults living with HIV in Senegal, the risk of hypertension was significantly higher in adults older than 50 years [6]. Several other studies corroborate that there is an increased risk of hypertension in HIV positive adults after the age of 40 [9,15,16]. The findings in this study broaden existing literature by revealing an expanded age of hypertension risk among PLHIV. Additionally, these results should broaden the scope of existing and new hypertension and HIV programmes which would have previously targeted older adults living with HIV given the literature of hypertension risk being prevalent only after the age of 40. However, in light of the younger HIV population group now also being at risk of hypertension, young adults diagnosed with HIV meet the inclusion criteria for HIV and hypertension prevention and treatment initiatives. Identifiably, there is a need to scale up early screening and treatment for hypertension among PLWH. By assessing the time to hypertension diagnosis among PLWH, the study further shows the period for patients and integrated HIV and hypertension programmes health programmes to prioritize prevention activities for hypertension.

The reason for the early age of hypertension risk could be as a result of early debut of chronic risk factors. For example, research shows that tobacco use often begins in the teenage years [14, 15, 16, 17, 19]. One study showed that over 30% of adolescent and young adults aged 12–26 and living with HIV smoked tobacco at least once a week [20]. Interestingly, no significant association was observed between chronic risk factors and hypertension development among PLWH in this study. Reasons for this could be adduced to that the NIDS survey used in this study relied on self-reported data of chronic risk behaviours such as smoking and alcohol. In studies where a high smoking prevalence was found, investigators used carbon monoxide tests, among others, to test for smoking [21]. Another plausible reason could be early initiation on antiretroviral treatment (ART). In South Africa, approximately 63% of children (0–14 years) are on ART [22]. These children are expected to be on treatment for their life time, at least up until an HIV cure is found. Several studies have suggested that longer duration of HIV infection and ART is associated with an increased risk of hypertension in adults [13, 23]. These studies have shown that metabolic syndrome (MS); a group of components such as excessive body fat and abnormal cholesterol; is one of the side-effects of ART [23, 24]. MS in tern increases the risk of chronic conditions such as hypertension, stroke and other cardiovascular diseases [25]. In one study, hypertension was found to be higher (28.7%) among ART treated patients as compared to ART naïve controls [26].

Some studies have shown that demographic and socio-economic factors such as age, gender, race, level of education and income are associated with hypertension in PLHIV [30]. For example a study in Ethiopia found that HIV diagnosed patients with some education and an average monthly income were significantly more likely to develop hypertension [31]. However, we found that while the hazard of hypertension was 0.22 and 0.59 times higher among male adults with HIV, these results were not significant. Similarly, we found no association between level of education, employment status or any of the demographic and socio-economic variables assessed in this study. The observed difference between our findings and previous studies could be due to differences in study design as the majority of the studies conducted were cross-sectional.

## 5 Limitations and strengths

This study has some important limitations. Firstly, the findings reported in this study are part of a larger mixed methods research in fulfilment of one of the authors doctoral studies and as a result, not representative of the full scope of the extensive research conducted on this topic. This paper is part of a series of publications emanating from the larger doctoral project.

Secondly, the NID survey did not have a variable that assessed ART use or adherence. In other words, it is unknown whether the study sample included in this study were on treatment or ART naïve. The potential limitation that this has is that several studies have shown that ART is associated with an increased risk of hypertension in adults [13, 23]. It is therefore unclear whether the 10.5% hypertension risk found among PLWH in this study was due to ART or other factors because the NID survey did not assess ART use. However, after identifying the lack of the ART variable in the NIDS dataset, the authors conducted a sub-qualitative study with a sample of HIV positive persons who were on ART to further explore the comorbidity of HIV and hypertension and found a similar prevalence of hypertension among patients on ART. Some of the findings of this research have been accepted for publication in the BMC Health Services Research Journal as of May 2021.

In addition, the time of diagnosis for HIV was not quantified in the survey, that is, the point at which the adults tested positive for HIV was not specified in the dataset. Given the longitudinal nature of the dataset, wave 1 (2008) was assessed as the time point in which all persons came into the study and presented with HIV. Only adults living with HIV were included at wave 1. The proceeding waves [3, 5] were then used to assess the development of hypertension.

A major strength of this study lies in its use of longitudinal and event history analysis to assess time to hypertension development among PLHIV. Through employing these methods, the study was able to retrospectively follow HIV infected persons for almost a 10-year period to assess risk of hypertension.

## 6 Conclusion and recommendations

There is an increased risk of hypertension among young adults living with HIV in South Africa. HIV infected youth can decrease the risk factors associated with hypertension through engaging in regular exercise, adhering to a healthy diet and limiting the use of tobacco and alcohol. Existing and new HIV and hypertension programmes should include young people into chronic disease prevention and treatment initiatives as this study has shown the risk of hypertension to begin from the age of 30. Additionally, this study places emphasises on the importance for persons living with HIV to routinely check for hypertension diagnosis. Early detection of hypertension among PLHIV can ensure that they are initiated on chronic treatment timeously which can reduce morbidity and mortality related to HIV and hypertension comorbidity and improve the overall quality of life and life expectancy of the HIV positive population [32].

## Declarations

### Author contribution statement

Motlatso Godongwana: Conceived and designed the experiments; Performed the experiments; Analyzed and interpreted the data; Wrote the paper.

Nicole De Wet-Billings: Conceived and designed the experiments; Contributed reagents, materials, analysis tools or data.

### Funding statement

This work was supported by National Institute for the Humanities and the Social Sciences (NIHSS).

### Data availability statement

Data will be made available on request.

### Declaration of interests statement

The authors declare no conflict of interest.

### Additional information

No additional information is available for this paper.
